# Understanding the impact of interruptions to HIV services during the COVID-19 pandemic: A modelling study

**DOI:** 10.1016/j.eclinm.2020.100483

**Published:** 2020-07-31

**Authors:** Britta L. Jewell, Jennifer A. Smith, Timothy B. Hallett

**Affiliations:** MRC Centre for Global Infectious Disease Analysis, Department of Infectious Disease Epidemiology, Imperial College London, London, UK

**Keywords:** HIV, Mathematical modelling, Antiretroviral therapy, COVID-19

## Abstract

**Background:**

There is concern that the COVID-19 pandemic could severely disrupt HIV services in sub-Saharan Africa. However, it is difficult to determine priorities for maintaining different elements of existing HIV services given widespread uncertainty.

**Methods:**

We explore the impact of disruptions on HIV outcomes in South Africa, Malawi, Zimbabwe, and Uganda using a mathematical model, examine how impact is affected by model assumptions, and compare potential HIV deaths to those that may be caused by COVID-19 in the same settings.

**Findings:**

The most important determinant of HIV-related mortality is an interruption to antiretroviral treatment (ART) supply. A three-month interruption for 40% of those on ART could cause a similar number of additional deaths as those that might be saved from COVID-19 through social distancing. An interruption for more than 6–90% of individuals on ART for nine months could cause the number of HIV deaths to exceed the number of COVID-19 deaths, depending on the COVID-19 projection. However, if ART supply is maintained, but new treatment, voluntary medical male circumcision, and pre-exposure prophylaxis initiations cease for 3 months and condom use is reduced, increases in HIV deaths would be limited to <2% over five years, although this could still be accompanied by a 7% increase in new HIV infections.

**Interpretation:**

HIV deaths could increase substantially during the COVID-19 pandemic under reasonable worst-case assumptions about interruptions to HIV services. It is a priority in high-burden countries to ensure continuity of ART during the pandemic.

**Funding:**

Bill & Melinda Gates Foundation.

**Research in context**

**Evidence before this study**

We searched PubMed for articles published before 14th May 2020 with the terms: “HIV”[Title] AND (“COVID-19″[Title] OR “SARS-CoV-2″[Title] OR “coronavirus”[Title])) AND “Africa”[All Fields]) AND (“Data”[Title] OR “Model”[Title]). No search results were returned. A recent editorial in *The Lancet HIV* [1] noted the growing concern about the impact of disruptions to HIV programs during the COVID-19 pandemic and the relevant policy developments that are in progress currently. In a ‘Feature’ article, *The Lancet HIV* describe disruptions that have already been reported anecdotally in TB programs [2].

**Added value of this study**

We provided a full modelling exploration of the potential impact of disruptions in four sub-Saharan countries, examine how this impact is affected by assumptions made in the model and make a comparison between the potential impact of the COVID-19 epidemic in the same settings.

**Implications of all the available evidence**

It is clear that the most important element of HIV programs to be maintained is the continuity of ART, although the potential impact of a disruptions is highly uncertain – due to not knowing the extent of any disruption and not knowing what the mortality risk is for those with ART interrupted. The impact could be very limited, but, under ‘reasonable worst-case assumptions,’ has the potential to be extremely high – even to the point of being comparable with the range of deaths projected under reasonable worst-case scenarios for COVID-19. This highlights how dependent so many persons are on ART programs in sub-Saharan Africa and the high priority that must be placed on ensuring that their treatment continues throughout the COVID-19 pandemic.

Alt-text: Unlabelled box

## 1 Introduction

The COVID-19 pandemic poses a great threat to the health of populations worldwide [3]. In addition to the direct health impact of the COVID-19 pandemic itself, there may also be a detrimental impact on service provision for other health conditions due to increasing demands on overall health service capacity, interruptions to supply of medicines, or funding shortages. This may be particularly detrimental for countries in sub-Saharan Africa that suffer from high burdens of other diseases, including HIV, tuberculosis, and malaria [4]. While no pandemic like COVID-19 has occurred in modern history, the Ebola epidemic in Guinea, for example, ultimately led to more deaths from malaria than those directly caused by Ebola, due to a lack of malaria treatment provision [5].

Early data from countries shows that there may be interruptions to HIV prevention programs, such as voluntary medical male circumcision (VMMC) and pre-exposure prophylaxis (PrEP) [2,6]. Interruptions to supply chains for antiretroviral therapy (ART) for people living with HIV (PLHIV) remains an additional possibility, which may have a substantial effect on health outcomes for PLHIV in sub-Saharan Africa.

Interruptions to different elements of the HIV treatment and prevention cascades are likely to have differential impacts on the resulting loss of health and it will be useful for decision-makers to understand the relative impact of different reductions in service to inform planning for service continuity during the COVID-19 pandemic. Here we use a mathematical model to examine the impact of potential hypothetical disruptions to HIV services in four countries in sub-Saharan Africa (South Africa, Malawi, Zimbabwe, and Uganda) and compare the resulting outcomes to projections for deaths that could arise from COVID-19 epidemics in the same settings.

## 2 Methods

We used an established deterministic mathematical model of the HIV epidemic in four countries with moderate to high HIV prevalence in sub-Saharan Africa – South Africa (20.4% prevalence [7]), Malawi (9.2% prevalence [7]), Zimbabwe (12.7% prevalence [7]) and Uganda (5.7% prevalence [7]) – to quantify the impact of theoretical disruptions to HIV services [8], [9], [10]. The number of ‘excess’ deaths that are attributed to these disruptions are computed over a five-year period, 2020–2025, in reference to a model projection in which no interruptions occur and coverage of programs expands in the manner that would have otherwise been anticipated.

First, we examined the individual effect of six different types of disruptions, each lasting for three months. A three-month duration was chosen as an illustrative scenario of potential disruptions but does not represent a prediction of how long disruptions will last in reality. The six types of disruptions considered as input assumptions were:

• ‘Reduced contact rates’: Universal 10% reduction in the formation rate of new sexual partnerships
• ‘No new VMMCs’: Cessation in programs implementing VMMC
• ‘Viral suppression decreases by 10%’: A 10% reduction in the proportion of those on ART that are virally suppressed
• ‘No new ART initiations’: Cessation in enrolment in ART programs for persons newly starting ART
• ‘Condom use reduced by 50%’: Condoms are used in 50% fewer sex acts
• ‘Interruption in ART for 40% of individuals’: 40% of those on ART are forced to temporarily discontinue ART

We then hypothesised three patterns of overall disruption to HIV services that might occur during the COVID-19 epidemic, incorporating all the individual elements reported above. These scenarios are described below and summarised in Table 1. Each scenario is cumulative in the sense that it incorporates the change described in all less severe scenarios. In the first instance, we assume the disruption begins in mid-2020 and lasts for three months, and that normal service resumes thereafter for all programs.

1. *Managed pause* (least severe):

In this scenario, the expansion of services planned for this time is paused but all services are maintained at their current levels. This might occur, for instance, if there is very little actual disruption to the HIV programs, but opportunities to expand are not available due to it being impractical to run outreach services and persons not yet engaged with care postpone testing.

2. *Managed disruption:*

In this scenario, substantial pressure on the health system and social distancing measures combine to disrupt services, but these are sufficiently well managed that their worst impacts are mitigated and the supply of ART is maintained. In addition to the effects of the first scenario (‘Managed pause’) there is also a reduction in condom use, as might be caused by reduced supply of condoms and less opportunity to acquire them, and decreased viral suppression, as might be caused by a combination of persons being less inclined or able to present for routine viral load testing, viral load testing being less widely available (due to increased usage of laboratory equipment), and short-term fluctuations in the supply of ART drugs.

3. *Interruption of supply* (most severe):

In this scenario, extreme pressure on the health system and/or strict interventions and stressed supply chains domestically and internationally combine to interrupt the supply of key medicines, with the result that a fraction of PLHIV on ART are temporarily forced off ART.

**Table 1:** Scenarios characterising disruption to HIV services due to the COVID-19 epidemic.

Scenario	Assumed Impact on HIV Programs
1. Managed pause (least severe)	-No new ART enrolments -No new VMMCs -(No new PrEP enrolments*) -Sexual contacts decrease by 10%
2. Managed disruption	-No new ART enrolments -No new VMMCs -(No PrEP enrolment or prescription refills*) -Viral suppression decreases by 10% across individuals on ART -Condom use decreases by 50% -Sexual contacts decrease by 10%
3. Interruption of supply (most severe)	-No new ART enrolments -No new VMMCs -(No PrEP enrolment or prescription refills*) -Viral suppression decreases by 10% across individuals on ART -40% of individuals on ART go off ART for duration of disruption, assuming a mean monthly mortality risk of 0.24% during the disruption⁎⁎ -Condom use decreases by 50% -Sexual contacts decrease by 10%

⁎ PrEP coverage is assumed to be very low and does not affect directly results presented here.

⁎⁎ We optimistically assume that if individuals have stopped ART due to the COVID-19 disruption, they immediately return to ART once supply resumes and their long-term prognosis on ART is not affected by the interruption in ART usage.

We compared the effect of each HIV disruption scenario on HIV programmes to the direct effects from a COVID-19 epidemic in each respective country. Three models providing publicly available COVID-19 mortality estimates for each country in the case of an unmitigated epidemic were found via a literature search. Modelled projections from Walker et al. [3] use three scenarios of epidemic spread, with the assumption that the basic reproduction number for COVID-19, R_0_, is 2.3 (Table 2). Results are also compared to projections from an online model by Pearson et al. [11] that used relatively similar assumptions, and a further model by Cabore et al., from authors at the WHO Regional Office for Africa, which uses different assumptions [12].

**Table 2:** Public health responses to the COVID-19 epidemic (from [3,11,12]).

	Scenarios from Walker et al. [3]	Scenarios from Pearson et al. [11]	Scenarios from Cabore et al. [12]
Unmitigated epidemic (most severe)*	No action is taken and R_0_ is 2.3.	No action is taken and R_0_ is 2.7.	No action is taken and R_0_ is 1.7 overall (range of 1.5–1.8, depending on country).
Social distancing	Population-level social distancing, i.e. the maximum reduction in the final scale of the epidemic that can be achieved through a uniform reduction in the rate at which individuals contact one another, short of suppression (for South Africa, e.g., this equates to a 37–48% reduction in social contacts).	Population-level social distancing, i.e. the maximum reduction in the final scale of the epidemic that can be achieved through a uniform reduction in the rate at which individuals contact one another, short of suppression (for all countries, this equates to a 20% reduction in social contacts).	Not modelled.
Enhanced social distancing interventions (least severe)	Population-level social distancing as above, with individuals aged 70 years old and older additionally reducing their social contact rates by 60%.	Shielding of 60% of those aged 60 years old and older with a 60% reduction in transmission in addition to a 20% reduction in contacts outside the household and a 25% reduction in transmission from symptomatic individuals.	Not modelled.

⁎ This is provided as a comparator ‘Reasonable Worst-Case Scenario’: it is considered very unlikely because, in reality, actions being taken to mitigate the epidemic would have an effect.

Results are sensitive to the assumed risk of dying for PLHIV who have had their ART supply interrupted. This quantity is not well known or understood as such a disruption has not ever happened to HIV health services. In the model, PLHIV who experience an ART interruption experience a risk of progressing to AIDS and a further risk of progressing to death, resulting in an average monthly mortality risk of 0.24% in the main analysis. We also conducted a sensitivity analysis using three plausible values for this parameter, as mortality in the case of a forced interruption of ART could be lower or higher than previously observed (Table 3).

**Table 3:** Alternative assumptions used for the risk of death experienced by those PLHIV whose ART supply is interrupted.

	*Average monthly mortality risk*	*Proportion that would die after one year*	*Justification*
*Lower bound*	0.10%	1.24%	This is a hypothetical best-case scenario in which the vast majority of individuals do not deteriorate rapidly.
*Medium*	0.24%	2.91%	The SMART trial found a 3% risk at 12 months of either death of an opportunistic infection for those with interrupted ART [13]. This also implies a mean survival time approximately equivalent to that for HIV-positive persons who have never been on ART [14].
*Upper bound*	0.44%	5.28%	This is the hypothetical worst-case scenario in which many persons deteriorate more rapidly.

## 3 Results

### 3.1 Changes in HIV incidence and mortality over five years

**Fig. 1:**
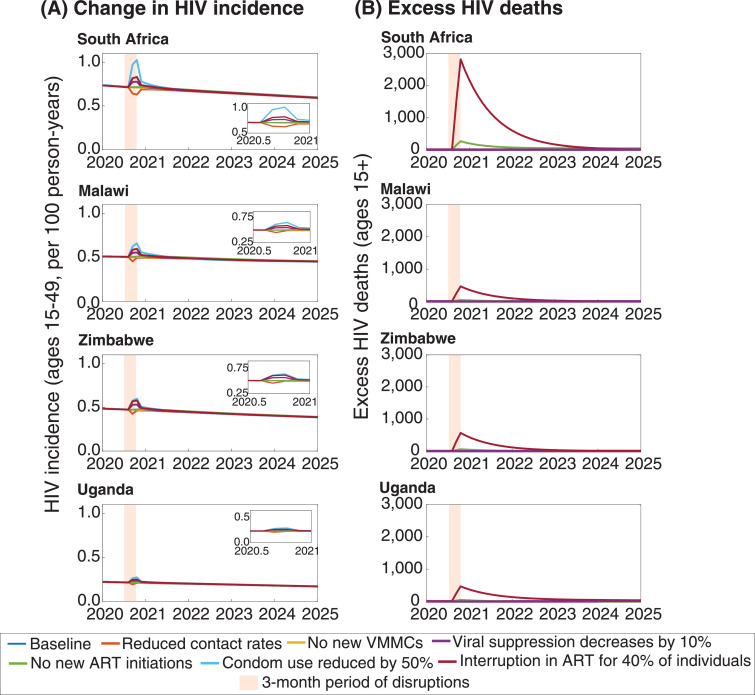
Potential changes in HIV incidence (A) and deaths (B) following a hypothetical three-month disruption of HIV services. Orange shading indicates the period during which the three-month disruptions occur. Baseline (no COVID-19 epidemic occurs) = dark blue line; Reduced contact rates = orange line; No new VMMCs = yellow line; Viral suppression decreases by 10% = purple line; No new ART initiations = green line; Condom use reduced by 50% = light blue line; Interruption in ART for 40% of individuals = dark red line. Panel insets in (A) show a magnified view of HIV incidence patterns from 2020.5 to 2021.

The individual effect on HIV incidence and mortality for each type of disruption in the four countries is shown in Fig. 1. In all settings, a decrease in sexual contact rates would result in a period of reduced HIV incidence, with incidence quickly returning to its previous levels soon after the disruption ends. In contrast, a 50% reduction in condom use would have the greatest effect on increasing incidence during the disruption. Reductions in viral suppression and interruptions in ART would also cause increases in incidence, though likely of a lesser magnitude. A temporary pause in the expansion of VMMC programmes would not be expected to have a large effect at this scale, in comparison.

For HIV deaths, an interruption in ART would cause a greater increase in HIV-related mortality than any of the other changes. There would continue to be additional HIV deaths in the years that follow the disruption because some of those whose ART was interrupted will have had their CD4 cells depleted to such an extent that they remain at a heightened risk of death.

South Africa would experience the largest changes in both HIV incidence and mortality as a result of the interruptions, as South Africa has both the highest incidence and greatest number of PLHIV on ART of any of the four countries represented. Furthermore, in South Africa, which is rapidly scaling up treatment programmes, there are also additional deaths caused by the temporary cessation in new treatment initiations. This is because a small fraction of those whose timely ART initiation is delayed until after the disruptions will have progressed to the point at which their CD4 count has fallen too low to be reconstituted or opportunistic infections have become unmanaged.

### 3.2 Comparison between COVID-19 and HIV deaths over five years

**Fig. 2:**
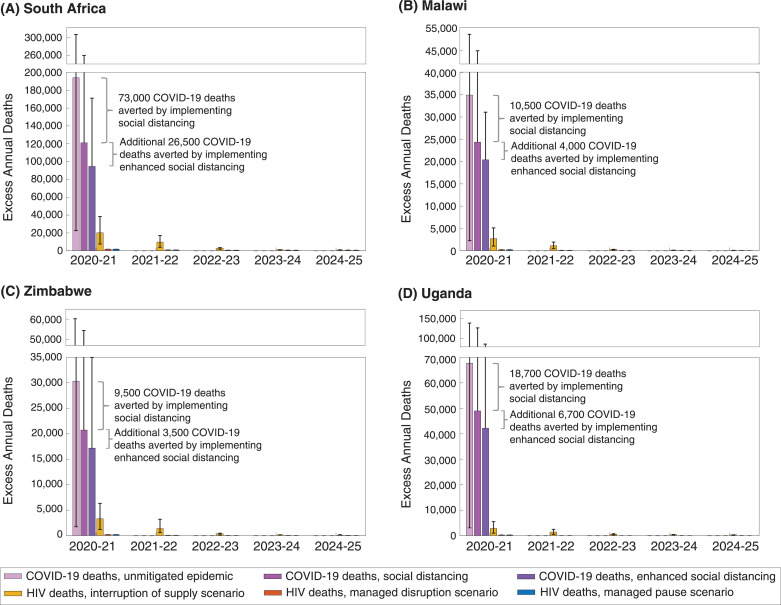
Projected direct and indirect deaths over time for South Africa (A), Malawi (B), Zimbabwe (C), and Uganda (D), under different assumptions about the COVID-19 epidemic in Walker et al. and the impact on HIV services (Table 1), for a three-month service interruption. The purple bars for COVID-19 deaths represent estimates from Walker et al. The whiskers represent estimates from Cabore et al. and Pearson et al. for an in an unmitigated epidemic, and Pearson et al. only for an epidemic with social distancing and enhanced social distancing from Table 2; no estimates from Cabore et al. were available for these scenarios [11,12]. Deaths averted by implementing social distancing and enhanced social distancing are from Walker et al. Yellow bars represent HIV deaths in the ‘interruption of supply’ scenario; red bars represent HIV deaths in the ‘managed disruption’ scenario; blue bars represent HIV deaths in the ‘managed pause’ scenario (all from Table 1). Confidence intervals for the HIV mortality scenarios represent uncertainty in the mortality risk for individuals with interrupted ART, ranging from a mean monthly mortality risk of 0.10% to 0.44%.

Fig. 2 shows the comparison between the projected COVID-19 deaths and the corresponding projections of additional HIV-related deaths caused by the patterns of disruptions assumed in the different scenarios in Table 1. In the projections, COVID-19 deaths are expected to be limited to the first year because the projections show the epidemic ending within that time, although it is possible that a mitigated epidemic would last longer than one year. This contrasts with the projections for HIV-related deaths, which continue to occur through 2025 and beyond.

For both the ‘Managed Pause’ and ‘Managed Disruption’ scenarios, excess mortality over five years is limited to, respectively, a 1 or 2% increase from the expected HIV-related mortality over this period. However, in the ‘Interruption of Supply’ scenario in which 40% of individuals are assumed to experience an interruption in ART supply for three months, the excess number of deaths caused over the five-year period is substantial. In all countries, this would be comparable to the number of COVID-19 deaths that are projected to be potentially averted by implementing social distancing or enhanced social distancing by the Walker et al. and Pearson et al. projections, and comparable to the total number of COVID-19 deaths in the Cabore et al. projections.

### 3.3 Sensitivity analysis for the duration of disruptions and the HIV-related mortality risk following ART interruption

**Fig. 3:**
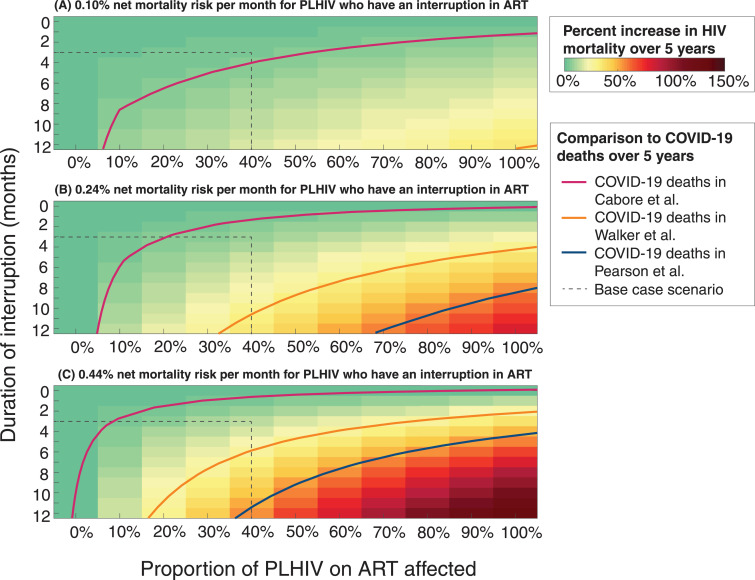
The excess HIV deaths over 5 years caused by the disruptions, as a percentage of the total number of HIV deaths expected without the disruptions. Results give the mean across the four countries considered – South Africa, Malawi, Zimbabwe, and Uganda. Plots shown give this metric with respect to the duration of interruption in months (vertical axes) and the proportion of people living with HIV (PLHIV) on antiretroviral therapy (ART) affected by the interruption (horizontal axes). The panels show the results under three different assumptions about the average net monthly mortality risk for those experience an interruption in ART supply: (A) 0.10%, (B) 0.24%, and (C) 0.44%. Contour lines show where the total number of deaths is greater than the deaths expected for COVID-19 epidemics, averaged across the four countries, for three different models of COVID-19 (Cabore et al., [12] Walker et al., [3] and Pearson et al. [11]). Light red lines correspond to the mean number of COVID-19 deaths in the four countries in Cabore et al.; orange and blue lines correspond to the mean number of COVID-19 deaths with enhanced social distancing in the four countries in Walker et al. and Pearson et al., respectively. Dashed lines correspond to the base case scenario of 40% of individuals experiencing a three-month interruption of ART.

Given that little data are available about the mortality risk for individuals who have an interruption in ART, and that the extent and duration of any disruption of ART is unknown, we modelled a range of potential durations of interruption, the proportion of PLHIV on ART affected, and the associated increase in HIV mortality over 5 years (Fig. 3). We also compared deaths in each scenario to the expected number of COVID-19 deaths occurring over the same period according to three different COVID-19 models (Walker et al. [3] and Pearson et al. [11], both with the assumption of enhanced social distancing interventions, and Cabore et al. [12] with the assumption of an unmitigated epidemic but a lower R_0_). This reveals that the relative scales of projected COVID-19 deaths and HIV deaths that might be caused by disruptions to services are both highly uncertain but overlap for plausible sets of assumptions.

Under the median mortality risk assumption, an interruption of ART lasting for nine months would lead to an additional number of HIV-related deaths equalling the total deaths caused by COVID-19 in these settings according to the Cabore et al. projections, if the interruption affected at least 6% of those currently on ART. If the comparison is made with the Walker et al. or Pearson et al. projections for COVID-19 deaths, then the same number of deaths would be caused if 47% or 90% of those on ART had their treatment interrupted, respectively. For the Cabore et al. projections, an interruption of ART for one month for at least 60% of PLHIV on ART could equal the total COVID-19 deaths, whereas for the Walker et al. and Pearson et al. projections, the minimum duration of an ART interruption to match the estimated number of COVID-19 deaths would be five and nine months, respectively.

For the lower assumed mortality risk for those off ART, the number of HIV-related deaths would increase by a maximum of 34% if all individuals stopped ART for 12 months, which would approximately equal the number of COVID-19 deaths with enhanced social distancing according to the Walker et al. model. For the higher assumed mortality risk, HIV-related mortality could reach a maximum 135% increase in over five years and the number of HIV-related deaths could exceed more than two times the number of COVID-19 deaths in the Pearson et al. model, five times the number of COVID-19 deaths in the Walker et al. model, and 29 times the number of COVID-19 deaths in the Cabore et al. model.

## 4 Discussion

Maintaining ART treatment during any health system disruptions that might occur as a result of the COVID-19 pandemic is the overriding priority for HIV programs. A wide-spread and long-lasting interruption to ART supply could cause additional deaths on the same order of magnitude as those which could be saved by interventions to mitigate the COVID-19 epidemic or match the deaths resulting from the COVID-19 epidemic itself. Some of the negative impacts of service disruption could be minimised by policy changes that are beneficial to HIV programs in the long-term – for example, adaptations to ART delivery, such as multi-month ART prescriptions or dispensation of medication outside of health facilities, are now recommended for consideration in appropriate circumstances by the WHO and PEPFAR [15,16]. However, if an interruption to ART supplies does occur, it may also take programs longer than assumed here to return to status quo, with further adverse effects expected among individuals who experience long-term ART disruptions.

Some short-term changes to programs, such as scaling back new VMMC procedures and reduced availability of condoms, may be less impactful in terms of the excess number of deaths in the medium-term, but new HIV infections could increase as a result and have further long-term ramifications not captured by the five-year time horizon. This would particularly true for disruptions lasting longer than three months. It is also notable that the number of life-years lost to each COVID-19 death may be fewer than those lost to HIV-related deaths, given the strong relationship between COVID-19 mortality and age [17,18]. The analysis presented here is for four countries with different population sizes and levels of HIV prevalence, but it is anticipated that the overall direction of the results will be similar for many countries in sub-Saharan Africa with large HIV epidemics. Another study comparing different disruptions to HIV services across five models found broadly similar results for an interruption of six months for 50% of those on ART in sub-Saharan Africa, while our results provide a complementary exploration of the uncertainty in such mortality estimates [19].

There are several important limitations to this analysis. First, we do not model any interaction between HIV or ART status and COVID-19 infection – that is, PLHIV are not assumed to be more or less likely to acquire or die from COVID-19. This may need to be revised as more information becomes available and could influence outcomes, particularly in areas with large numbers of PLHIV who are not virally suppressed. Second, the effect of a disruption on the risk of mother-to-child transmission is not included in this model and could result in further deaths and infections not represented here. Third, possible increases in drug resistance due to ART regimens being disrupted are not included, but these could also contribute to excess HIV deaths in the longer term [19]. Fourth, the monthly risk of death is likely to increase over time as individuals accrue time off ART; however, this is not represented in the model. In addition, there may be a great degree of heterogeneity among individuals experiencing an interruption of ART, with those having a history of low CD4 counts and opportunistic infections more likely to have a substantially higher mortality risk than those with many years of ART or high CD4 counts. Finally, disruptions could last longer than represented here and if this were to result in defunding of HIV programmes, the outcomes could be even more catastrophic.

We have also assumed a universal reduction in sexual risk behaviour during the interruption, but any changes in sexual risk behaviour as a result of COVID-19 interventions are not currently known. A reduction in sexual contacts could reduce incidence rates and hence new infections but would have a limited impact on the number of deaths over five years. Furthermore, interruptions to condom supplies could counteract any incidence decline. Reductions in condom availability could also have differential impacts on new HIV infections among different populations; for example, female sex workers might be at higher excess risk than women in stable partnerships. Ensuring support for community prevention programs during the COVID-19 epidemic will help to mitigate these potential effects.

There is also a high degree of uncertainty surrounding the extent of mortality due to COVID-19 in African countries. Three models have released projections of estimated deaths caused by COVID-19 in each country [3,11,12]. A model by Cabore et al. [12] from the WHO Regional Office for Africa projects substantially lower mortality (75–94% lower than the Walker et al. projections and 86–97% lower than the Pearson et al. projections with enhanced social distancing for the four countries in this analysis), due to an assumed lower risk of transmission compared to the other models. The relative strengths of the modelling approaches may be debated, but these varied projections show the range of impacts that are being discussed and such context is important: if COVID-19 deaths are closer to those predicted by Cabore et al., even a small proportion of individuals experiencing an interruption in ART, or a larger proportion for a short period of time, could lead to more deaths than from COVID-19 itself. Furthermore, HIV deaths resulting from an interruption in supply could exceed the number of COVID-19 deaths averted by implementing social distancing in both Walker et al. and Pearson et al. It is also possible that the number of COVID-19 deaths occurring beyond 2020–21 could be higher than estimated by these models if a vaccine or effective therapeutic is not found or if suppression strategies delay an epidemic to a later time period.

The assumptions made in this analysis are not predictions of the future, but hypothetical scenarios designed to highlight the importance of maintaining different services during COVID-19 epidemics. The model itself has many limitations, as noted above. Outside of these, the major uncertainties in this analysis can be classified into four groups: (1) uncertainty about the scale of the COVID-19 epidemic (both how far and fast will it spread and with what probability of death for those infected); (2) uncertainty into the extent to which HIV programs will actually be disrupted by the COVID-19 epidemic and the response to it; (3) uncertainty about changes to patterns of sexual risk behaviour in response to the epidemic; and (4) uncertainty about the mortality risk of those persons on ART who may suffer an interruption in the supply of drug. The range of mortality effects due to ART disruption will depend on the health and immune status of the person, the drugs they are using and their treatment history, as well as any steps that are taken to prolong supply (e.g., alternate day dosing). It is also possible that interruptions to services could be handled in a more nuanced way than assumed here, for example by prioritising maintenance of ART for those with treatment histories that indicate a greatest risk of opportunistic infections or death. For these reasons, we have repeated the analysis for a wide range of values, but even within this, it may be that the effect has been over- or under-estimated. Empirical data collection would help refine our analysis further, particularly data on ART stock levels in-country, anticipated supply chain issues, and the mortality risk for individuals experiencing time off ART.

This analysis shows that the impact of disruptions to HIV services during the COVID-19 pandemic could be limited, if countries are able to maintain key services, but under worst-case assumptions could also be comparable with the range of deaths projected to be caused by COVID-19. Ultimately, ensuring the ART supply for individuals currently on treatment would minimise excess mortality among PLHIV in sub-Saharan Africa and should be a key priority for policymakers.

## Declaration of Competing Interest

BLJ reports personal fees from Kaiser Permanente outside the submitted work. JAS reports personal fees from the Bill and Melinda Gates Foundation and grants from USAID and the Bill and Melinda Gates Foundation outside the submitted work. TBH reports personal fees from The Global Fund, WHO and Bill & Melinda Gates Foundation outside the submitted work.

## Appendix Supplementary materials

Image, application 1
