# Supplementary material for: Understanding the impact of interruptions to HIV services during the COVID-19 pandemic: A modelling study
Source: eClinicalMedicine. 2020 Jul 31;26:100483. doi: 10.1016/j.eclinm.2020.100483 (PMC7564522; doi:10.1016/j.eclinm.2020.100483)
Supplement: Supplementary file 1 [file mmc1.docx]

Understanding the Impact of Interruptions to HIV Services During the COVID-19 Pandemic: A Modelling Study

**Supporting Information**

***Contents***

1. *Epidemic model of HIV transmission*
2. *Mortality risk sensitivity analyses*
3. *References*

***Overview***

*The COVID-19 pandemic may cause disruptions to many health services in sub-Saharan Africa, including those for the prevention and treatment of HIV. There are many dimensions of uncertainty surrounding the potential impact of such disruptions, including the duration of the disruption, the proportion of the population affected, the types of services affected, and the extent to which disruptions may affect HIV incidence and mortality. To address the potential impact of a number of disruptions, we used a dynamic HIV transmission model to simulate for scenarios of potential disruptions to HIV services and evaluated resulting health outcomes over time.*

1. Epidemic Model of HIV Transmission

# 1.1 Model overview

The model, adapted from a well-established model developed by Cremin, Smith, Beacroft, and colleagues (1-3), is a deterministic compartmental model defined by a set of ordinary differential equations. It is designed to represent heterosexual HIV transmission and contraceptive use at the population level and is used in this analysis to estimate the effect of potential interruptions to HIV services arising from the COVID-19 epidemic. Our aim is to estimate how these interruptions may affect HIV outcomes over time in four different settings in sub-Saharan Africa: South Africa, Malawi, Zimbabwe, and Uganda. The basic model structure remains the same for all four countries modelled, with adaptations to key parameters to represent each country’s respective demographics and HIV epidemic over time.

The model population is divided into compartments that are distinguished by sex, circumcision status (if male), age, infection stage, sexual behaviour and contraceptive use, with events (e.g. HIV infection, death, ART initiation etc.) represented as movement between these compartments (4-6). Heterogeneity in sexual behaviour is incorporated in the model by stratifying men and women into three risk groups according to their average effective partnership formation rate.

A full description of the model structure, parameter values used, and calibration is provided under the following sections: 1.2 Natural history of HIV infection, 1.3 Demography, 1.4 HIV transmission and sexual mixing, 1.5 Male circumcision, 1.6 Antiretroviral treatment, and 1.7 Model calibration.

# 1.2 Natural history of HIV infection

A flow diagram for the natural history of HIV infection and initiation on ART is shown in Figure S1.


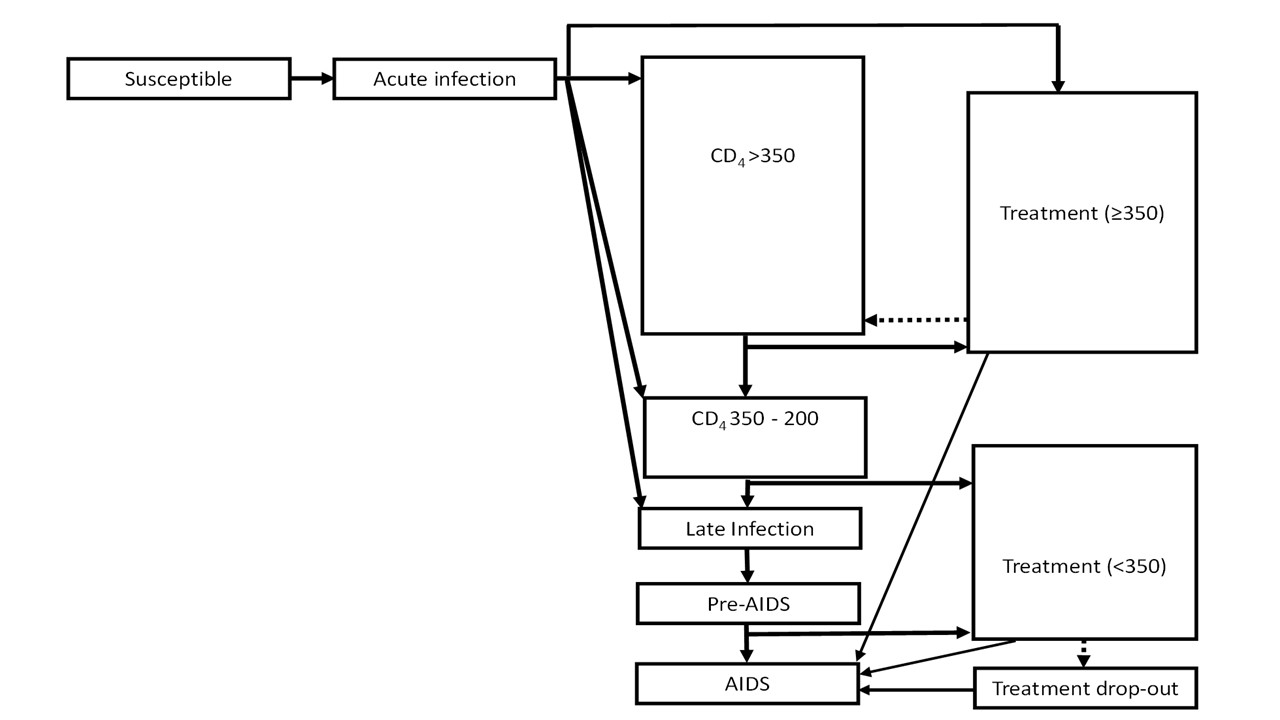


***Figure S1****: Natural history of HIV infection and ART initiation as represented in the model. Movement between compartments is indicated by arrows. Dotted arrows represent dropout from treatment compartments.*

The model is specified by the following ordinary differential equations:

The equations describing susceptible individuals are:

$\frac{dX_{l,k,1}^{0,p}}{dt}=\mu N\Psi_{\left( l,k,p,1 \right)}-\left( \lambda_{l,k,1}^{p}+\mu_{k,1} \right)X_{l,k,1}^{0,p}+f\left( 1 \right)+g(k)+ q(p)$ for a=1

$\frac{dX_{l,k,a}^{0,p}}{dt}=-\left( \lambda_{l,k,A}^{p}+\mu_{k,a} \right)X_{l,k,a}^{0,p}+f\left( a \right)+g(k)+ q(p)$ for a≠1

(1)

The equations describing HIV infected individuals are:

$$\frac{dX_{l,k,a}^{1,p}}{dt}=\lambda_{l,k,A}^{p}X_{l,k,a}^{0,p}-\left( {(\gamma1+\gamma2+\gamma3)\sigma}_{1}+\mu_{k,a} \right)X_{l,k,a}^{1,p}+f\left( a \right) + g(k)+ q(p)$$

$$\frac{dX_{l,k,a}^{2,p}}{dt}=\left( 1-\phi_{1} \right)\sigma_{1}{\gamma1X}_{l,k,a}^{1,p}+\zeta_{E}X_{l,k,a}^{7,p}-\left( \sigma_{2}+\mu_{k,a} \right)X_{l,k,a}^{2,p}+f\left( a \right) + g(k)+ q(p)$$

$$\frac{dX_{l,k,a}^{3,p}}{dt}=\left( 1-\phi_{2} \right)\sigma_{2}\gamma2X_{l,k,a}^{2,p}-\left( \sigma_{3}+\mu_{k,a} \right)X_{l,k,a}^{3,p}+f\left( a \right)+ g(k)+ q(p)$$

$$\frac{dX_{l,k,a}^{4,p}}{dt}=\left( 1-\phi_{3} \right)\sigma_{3}\gamma3X_{l,k,a}^{3,p}-\left( \sigma_{4}+\mu_{k,a} \right)X_{l,k,a}^{4,p}+f\left( a \right)+ g(k)+ q(p)$$

$$\frac{dX_{l,k,a}^{5,p}}{dt}=\sigma_{4}X_{l,k,a}^{4,p}-\left( \sigma_{5}+\mu_{k,a} \right)X_{l,k,a}^{5,p}+f\left( a \right)+ g(k)+ q(p)$$

$$\frac{dX_{l,k,a}^{6,p}}{dt}=\left( 1-\phi_{4} \right)\sigma_{5}X_{l,k,a}^{5,p}+\omega_{E}X_{l,k,a}^{7,p}+\omega_{L}X_{l,k,a}^{8,p}+\tau_{D}X_{l,k,a}^{9,p}-\left( \Omega+\mu_{k,a} \right)X_{l,k,a}^{6,p}+f\left( a \right)\ldots+ g\left( k \right)+ q(p)$$

$$\frac{dX_{l,k,a}^{7,p}}{dt}={\phi_{3}\sigma}_{3}X_{l,k,a}^{3,p}+{\phi_{4}\sigma}_{5}X_{l,k,a}^{5,p}-\left( \omega_{L}+\zeta_{L}+\mu_{k,a} \right)X_{l,k,a}^{7,p}+f\left( a \right)+ g(k)+ q(p)$$

$$\frac{dX_{l,k,a}^{8,p}}{dt}={\phi_{1}\sigma}_{1}\gamma1X_{l,k,a}^{1,p}+{\phi_{2}\sigma}_{2}X_{l,k,a}^{2,p}-\left( \omega_{E}+\zeta_{E}+\mu_{k,a} \right)X_{l,k,a}^{8,p}+f\left( a \right)+ g(k)+ q(p)$$

$$\frac{dX_{l,k,a}^{9,p}}{dt}=\zeta_{L}X_{l,k,a}^{8,p}-\left( \tau_{D}+\mu_{k,a} \right)X_{l,k,a}^{9,p}+f\left( a \right)+ g(k)+ q(p)$$

(2)

For a given stage of HIV infection, hazards of progression to the next stage are given by the rates σ _(1,2,3,4,5,6)_. The late infection stage is defined by the mean time between when CD4 count falls below 200 cells/μl and viremic rebound 19 months, on average, before death. The pre-AIDS stage characterizes the 9 month period of heightened infectiousness before AIDS, which represents a 10 month period of no transmission risk (7).

Mortality in the AIDS stage is denoted by the parameter Ω. An important limitation of this model is that AIDS-related mortality only applies to this final AIDS stage. However, in reality some infected individuals may die of AIDS-related illnesses at higher CD4 counts.

Several representations of ART initiation are possible in the model; ART can be initiated following acute infection, when an individual’s CD4 count drops below 350, 200, or 100 cells per microliter. The proportion of individuals initiating ART following acute infection, at CD4 <350 cells/μl, CD4 <200 cells/μl and CD4 <100 cells/μl are controlled by the parameters *ϕ_1,_ ϕ_2_*, *ϕ_3_* and *ϕ_4,_* respectively*_._* ART initiation at low CD4 counts (< 100 cells/μl) is used to represent the initial pattern of ART initiation (i.e. for urgent clinical need), when ART was first introduced.

ART is assumed to extend the survival of treated individuals (the increase in life expectancy depends on whether ART is initiated ‘≥350 cells/μl’ or ‘<350 cells/μl’) while reducing infectiousness (8, 9). Individuals initiating ART ≥350 cells/μl or <350 cells/μl are assumed to survive on average $\frac{1}{\omega_{E}}$ or $\frac{1}{\omega_{L}}$ years before progressing to AIDS, respectively. Drop outs from treatment initiated ≥350 cells/μl return to having a CD4 ≥350 cells/μl and progress through infection. Drop outs from treatment when initiated <350 cells/μl progress to AIDS after a period of slightly reduced infectiousness (resulting from an assumed period of time in which individuals have reduced viral load as they come off ART), represented by the ‘Treatment drop-out’ compartment in Figure S1.

| **Parameter** | **Symbol** | **Value** | **Source** |
| --- | --- | --- | --- |
| Mean duration of acute infection | 1/σ_1_ | 0.25 years | (7) |
| Mean duration from the end of acute infection to CD4 350 cells/μl | 1/σ_2_ | 9.25 years |  |
| Mean duration from CD4 350 cells/μl to CD4 200 cells/μl | 1/σ_3_ | 3.54 years |  |
| Mean duration from CD4<200 cells/μl to viremic rebound | 1/σ_4_ | 1.12 years |  |
| Mean duration of viremic rebound before AIDS | 1/σ_5_ | 0.75 years |  |
| Mean duration from treatment dropout to AIDS for individuals dropping off ART | 1/σ_6_ | 13 years | (10) |
| AIDS mortality rate | Ω | 1/0.833 (10 month period before death) |  |
| Proportion of HIV infected individuals starting infection at CD4> 350 cells/μl | $\gamma1$ | 0.81  (0.58+0.23) | (11) |
| Proportion of HIV infected individuals starting infection at CD4 200-350 cells/μl | $\gamma2$ | 0.16 |  |
| Proportion of HIV infected individuals starting infection at CD4<200 cells/μl | $\gamma3$ | 0.03 |  |

***Table S1:*** *Natural history of infection parameters.*

# Demography

The model is stratified by one-tenth of a single year of age from birth to 100 years. Ageing of individuals is represented by:

$$X_{l,k,1}^{0,p}= X_{l,1,a}^{s,p}b_{a} for a=1$$

$$X_{l,k,a}^{s,p}=X_{l,k,a-1}^{s,p} for a>1$$

(3)

$\Psi_{\left( l,k,p,a \right)}$ is the matrix of population distribution in the year the epidemic starts (*t_0_*) over each

l, k, a stratum and it is defined in terms of: (i) $\varphi_{f(l)}$ and $\varphi_{m(l)}$which are the proportion of females and males respectively in each risk activity group; and (ii) *f_a_* which is the proportion of the population in each year of age, with $\sum_{a=1}^{100} f_{a}=1$. The parameter *f_cm_* gives the fraction of males who are circumcised.

The total number in the population (N) and $\Psi_{\left( l,k,p,a \right)}$ are given by:

$$N=\sum_{l=1}^{3} \sum_{k=1}^{3} \sum_{p=1}^{7} \sum_{a=1}^{100} \left( X_{l,k,a}^{0,p}+X_{l,k,a}^{1,p}+X_{l,k,a}^{2,p}+X_{l,k,a}^{3,p}+X_{l,k,a}^{4,p}+X_{l,k,a}^{5,p}+X_{l,k,a}^{6,p}+X_{l,k,a}^{7,p}+X_{l,k,a}^{8,p}+X_{l,k,a}^{9,p} \right)$$

(4)

$$\Psi_{\left( l,k,p,a \right)}=\left\{ \begin{aligned} \frac{1}{2}\varphi_{f(l)}f_{p} f_{a} any l; k=1;any p; any a; \\ \frac{1}{2}\varphi_{m(l)}f_{a}\left( 1-f_{cm} \right) any l; k=2; p=1; any a; \\ \frac{1}{2}\varphi_{m(l)}f_{a}f_{cm} any l; k=3;p=1; any a; \end{aligned} \right.$$

(5)

Individuals enter the population as susceptible at birth (i.e. age zero), the distribution of whom is defined by the population distribution matrix over each *l, k and p* stratum ($\Psi_{\left( l,k,p,1 \right)}$), given by:

$$\Psi_{\left( l,k,1,1 \right)}=\left\{ \begin{aligned} \frac{1}{2}\varphi_{f\left( l \right)} any l; k=1;p=1 (no method); a=1 (0 years); \\ \frac{1}{2}\varphi_{m\left( l \right)}\left( 1-f_{cm} \right) any l; k=2;p=1 (no method); a=1 (0 years); \\ \frac{1}{2}\varphi_{m\left( l \right)}f_{cm} any l; k=3; p=1 (no method); a=1 (0 years); \end{aligned} \right.$$

(6)

Age-specific fertility rates (*b_a,t_*) and age- and sex-specific non-AIDS mortality rates (*μ_k,a,t_*) are taken from the UN World Population Prospects and are updated each year from the beginning of the epidemic to 2025 as estimated by that model (12). AIDS-related mortality is modelled explicitly (***Table*** S1). The population distribution by age at the beginning of the epidemic (*f _a_*) is taken from the same source (12).

# 1.4 HIV transmission and sexual mixing

##### *Force of infection*

The force of infection is the per capita rate at which susceptible individuals acquire infection. Following previous work [3], the per capita force of infection $\lambda_{l,k,A}^{p}$ is the force of infection experienced by individuals of each contraceptive group, risk group, sex, circumcision status (if male) and five year age group from the infected population of the opposite sex at a given time. Characteristics of an individual (p, l, k and A (where A is five year age group)) are distinguished from those of their sexual partners by means of a prime (i.e. p’, l’, k’ and A’). The force of infection is calculated by five-year age group and then applied to each single year of age in that group.

The force of infection depends on the pattern of partnership formation between different risk and five year age groups and on the probability of transmission per partnership, and is defined as:

For women:

$$\lambda_{l,1,A}^{p}=\sum_{l'} \sum_{k'} \sum_{p'} \sum_{A'} \sum_{s'} \left[ C_{g,A,l}\rho_{g,A,l,A^{'},l^{'}}\left( \frac{X_{l^{'},k^{'},A^{'}}^{s^{'}, p^{'}}}{\sum_{k^{'}=2}^{3} \sum_{p'} \sum_{s'} X_{l^{'},k^{'},A^{'}}^{s^{'},p^{'}}} \right)Z_{l,1,p,s^{'},l^{'},k^{'},p^{'}} \right]$$

For men:

$$\lambda_{l,k,A}^{p}=\sum_{l'} \sum_{p'} \sum_{A'} \sum_{s'} \left[ C_{g,A,l}\rho_{g,A,l,A^{'},l^{'}}\left( \frac{X_{l^{'},1,A'}^{s^{'}, p^{'}}}{\sum_{p'} \sum_{s'} X_{l^{'},1,A^{'}}^{s^{'},p^{'}}} \right)Z_{l,k,p,s^{'},l^{'},1,p^{'}} \right]$$

(7)

##### *Probability of transmission per partnership*

The probability of transmission per partnership depends on (i) the probability of transmission per sex act, and (ii) the number of sex acts during the partnership (which depends on the risk group of each partner). The probability of transmission per sex act depends on an individual’s circumcision status (if male), in addition to their partner’s state of HIV infection (including ART use), circumcision status (if male), and the degree of condom use in the partnership (which depends on the risk group of each partner).

A baseline transmission probability from uncircumcised males to females is assumed (β_0_). The difference in acquisition and transmission per sex act for other factors (e.g. stage of infection) is specified with respect to this baseline transmission probability using a multiplicative factor. The probability of HIV transmission per sex act is given by $\beta_{p,k}^{s^{'}k^{'}}$and depends on: s’ (partner’s HIV status), k’ (partner’s circumcision status (if male)), p (individual’s contraception status) and k (individual’s circumcision status (if male)). The probability of transmission from males to females is assumed to be identical to that for transmission from females to males. Male circumcision is assumed to reduce the risk of acquisition but not onward transmission.

The number of sex acts in a partnership depends on the risk group of both partners and is given by the matrix *n_sex_(l,l’)*. Condom use is modeled as a proportion of sex acts in which condoms are used via the matrix *CU(l,l’)*, which defines condom use in a partnership between an individual’s risk group l and their partner’s risk group l’, modulated by any increase in condom use due to changes over time $\bar{q}$(t). The efficacy of condoms is given as *ϖ*.

The probability of transmission per partnership $Z_{l,k,p,s^{'},l^{'},k^{'},p^{'}}$ is defined as:

For women and for uncircumcised men:

$$Z_{l,k,1,s^{'},l^{'},1,p^{'}}=1-\left( \left( 1-\beta_{1,k}^{s^{'}k^{'}}{\varpi)}^{X} \right)\left( (1-\beta_{1,k}^{s^{'}k^{'}})^{\bar{X}} \right. \right)$$

For circumcised men:

$$Z_{l,3,1,s^{'},l^{'},k^{'},p^{'}}=1-\left( \left( 1-\beta_{1,3}^{s^{'}k^{'}}{\varpi)}^{X} \right)\left( (1-\beta_{1,3}^{s^{'}k^{'}})^{\bar{X}} \right. \right)$$

(8)

Where:

$$X=CU(l,l^{'})\bar{q}_{(t)}n_{sex}\left( l,l^{'} \right)$$

$$\bar{X}=\left( 1-CU(l,l^{'})\bar{q}_{\left( t \right)} \right)n_{sex}\left( l,l^{'} \right)$$

(9)

That is, *X* is the number of sex acts protected by condoms in a partnership between an individual of risk group *l* and their partner of risk group *l’* and $\bar{X}$ is the number of sex acts not protected by condoms in a partnership between an individual of risk group *l* and their partner of risk group *l’*.

##### *Sexual mixing*

The mixing pattern is defined with respect to sex, five-year age group and behavioural risk group. The proportion of sexual partnerships that an individual of sex g (where g=1 refers to females and g=2 to males), 5 year age group A and risk group *l* forms with an individual of the opposite sex, age group A*’* and risk group *l’*, is given by $\rho_{g,A,l,A^{'}l^{'}}$, and is defined as:

$$P_{1,A,l,A^{'}l^{'}}=\varepsilon_{A}\varepsilon_{l}\left( \delta_{A,A^{'}}\delta_{l,l^{'}} \right)+\left( 1-\varepsilon_{A} \right)\varepsilon_{l}\left( \delta_{l,l^{'}}\frac{C_{2,A^{'},l^{'}}\sum_{k^{'}=2}^{3} \sum_{p^{'}} \sum_{s^{'}} X_{l^{'},k^{'},A^{'}}^{s^{'},p^{'}}}{\sum_{A^{'}} C_{2,A^{'},l^{'}}\sum_{k^{'}=2}^{3} \sum_{p^{'}} \sum_{s^{'}} X_{l^{'},k^{'},A^{'}}^{s^{'},p^{'}}} \right)+\varepsilon_{A}\left( 1-\varepsilon_{l} \right)\left( \delta_{A,A^{'}}\frac{C_{2,A^{'},l^{'}}N_{g^{'}}\left( A^{'},l^{'} \right)}{\sum_{l^{'}} C_{2,A^{'},l^{'}}\sum_{k^{'}=2}^{3} \sum_{p^{'}} \sum_{s^{'}} X_{l^{'},k^{'},A^{'}}^{s^{'},p^{'}}} \right)+\left( 1-\varepsilon_{A} \right)\left( 1-\varepsilon_{l} \right)\left( \frac{C_{2,A^{'},l^{'}}N_{g^{'}}\left( A^{'},l^{'} \right)}{\sum_{A^{'}} \sum_{l^{'}} C_{2,A^{'},l^{'}}\sum_{k^{'}=2}^{3} \sum_{p^{'}} \sum_{s^{'}} X_{l^{'},k^{'},A^{'}}^{s^{'},p^{'}}} \right)$$

$$P_{2,A,l,A^{'}l^{'}}=\varepsilon_{A}\varepsilon_{l}\left( \delta_{A,A^{'}}\delta_{l,l^{'}} \right)+\left( 1-\varepsilon_{A} \right)\varepsilon_{l}\left( \delta_{l,l^{'}}\frac{C_{1,A^{'},l^{'}}\sum_{p^{'}} \sum_{s^{'}} X_{l^{'},1,A^{'}}^{s^{'},p^{'}}}{\sum_{A^{'}} C_{1,A^{'},l^{'}}\sum_{p^{'}} \sum_{s^{'}} X_{l^{'},1,A^{'}}^{s^{'},p^{'}}} \right)+\varepsilon_{A}\left( 1-\varepsilon_{l} \right)\left( \delta_{A,A^{'}}\frac{C_{1,A^{'},l^{'}}\sum_{p^{'}} \sum_{s^{'}} X_{l^{'},1,A^{'}}^{s^{'},p^{'}}}{\sum_{l^{'}} C_{1,A^{'},l^{'}}\sum_{p^{'}} \sum_{s^{'}} X_{l^{'},1,A^{'}}^{s^{'},p^{'}}} \right)+\left( 1-\varepsilon_{A} \right)\left( 1-\varepsilon_{l} \right)\left( \frac{C_{1,A^{'},l^{'}}\sum_{p^{'}} \sum_{s^{'}} X_{l^{'},1,A^{'}}^{s^{'},p^{'}}}{\sum_{A^{'}} \sum_{l^{'}} C_{1,A^{'},l^{'}}\sum_{p^{'}} \sum_{s^{'}} X_{l^{'},1,A^{'}}^{s^{'},p^{'}}} \right)$$

Note: $\sum_{A^{'}} \sum_{l^{'}} \rho_{g,A,l,A^{'},l^{'}}=1$

(10)

The parameter *C_g,A,l_* gives the mean number of partners in a year per individual of sex *g* in age group A and risk group *l*. The degree of assortativity in mixing with respect to age and with respect to risk group are given by ε_A_ and ε_l_, respectively. The identity matrix with respect to risk is given by *δ_l,l’_* whereby:

$$\delta_{l,l^{'}}=\left\{ \begin{aligned} 1, if l=l^{'} \\ 0, if l\neq l^{'} \end{aligned} \right.$$

(11)

A discrepancy matrix $D_{A_{2},l_{2},A_{1},l_{1}}$is defined to balance the number of sexual partnerships between males and females formed with respect to each age group and risk group, where *A_2_* and *l_2_* are the age and risk group of the male partner and *A_1_* and *l_1_* are the age and risk group of the female partner. It is calculated as follows:

$$D_{A_{2},l_{2},A_{1},l_{1}}= \frac{\rho_{2,A,l,A^{'}l^{'}}C_{2,A,l}\sum_{k=2}^{3} \sum_{p} \sum_{s} X_{l,k,A}^{s,p}}{\rho_{1,A,l,A^{'}l^{'}}C_{1,A,l}\sum_{p} \sum_{s} X_{l,1,A}^{s,p}}$$

(12)

The extent to which balancing of the number of sexual partnerships is male-driven is determined by parameter *θ*. When *θ=0.5* the sexes compromise equally. Balancing the number of sexual partnerships is carried out with respect to both partners’ age and risk groups and is represented by:

$$\rho_{2,A,l,A^{'}l^{'}}\longrightarrow D_{A_{2},l_{2},A_{1},l_{1}}{}^{(\theta-1)}{\rho_{2,A,l,A^{'}l^{'}}}$$

$$\rho_{1,A,l,A^{'}l^{'}}\longrightarrow D_{A_{2},l_{2},A_{1},l_{1}}{}^{(\theta)}{\rho_{1,A,l,A^{'}l^{'}}}$$

(13)

| **Parameter** | **Symbol** | **Value** | **Notes** |
| --- | --- | --- | --- |
| Fraction of women in “low” risk group | *ψf(1)* | 0.09-0.25 | Calibrated separately for each modelled country |
| Fraction of women in “medium” risk group | *ψf(2)* | 0.17-0.71 | Calibrated separately for each modelled country |
| Fraction of men in low risk group | *ψm(1)* | 0.08-0.32 | Calibrated separately for each modelled country |
| Fraction of men in medium risk group | *ψm(2)* | 0.64-0.68 | Calibrated separately for each modelled country |

*Table S2: Behavioural parameters and values. Remaining women or men not in the low or medium risk groups are assumed to be high risk.*

The number of sex acts per partnership depends on behavioural risk group. The “low” risk groups are intended to reflect long-term stable partnerships and these are assumed to have a high number of sex acts overall. A value of 100 sex acts each year is assumed based on reported frequency of sex in marital relationships in Southern Africa [14]. Those in the higher risk groups tend to form more partnerships, but each of these partnerships comprises fewer sex acts and higher condom use. A value of two sex acts is assumed as a representative assumption of casual and commercial sex.

| **Parameter** | **Symbol** | **Value** | **Source** |
| --- | --- | --- | --- |
| Baseline transmission probability from uncircumcised males in the asymptomatic stage of HIV infection to females in a single act of unprotected sex | β_0_ | β_0 =_ 0.0008-0.0023 | Calibrated for each modelled country.  The parameter is representative and captures impact of other risk factors not explicitly models such as infection with STIs other than HIV (13, 14). |
| **Factor increase in transmission:** |  |  |  |
| To users of DMPA-IM | $\beta_{3,1}^{s,k^{'}}$ | 1 | (15) |
| From population with acute HIV infection | $\beta_{p,k}^{1,k^{'}}$ | 27 | (7) |
| From population with chronic HIV infection and CD4 >350 cells/μL | $\beta_{p,k}^{2,k^{'}}$ | 1 | The baseline transmission probability is assumed to apply from the end of acute infection until the period of heightened infectiousness 19-10 months before death (7). |
| From population with chronic HIV infection and CD4 >200 cells/μL but <350 cells/μL | $\beta_{p,k}^{3,k^{'}}$ | 1.6 |  |
| From population in late infection | $\beta_{p,k}^{4,k^{'}}$ | 3.8 | (7) |
| From population in pre-AIDS | $\beta_{p,k}^{5,k^{'}}$ | 3.8 |  |
| From population in AIDS | $\beta_{p,k}^{6,k^{'}}$ | 3.8 |  |
| From population on early ART | $\beta_{p,k}^{8,k^{'}}$ | 0.08 | (8) |
| From population on late ART | $\beta_{p,k}^{10,k^{'}}$ | 0.08 | (8) |
| From population who have dropped out of ART | $\beta_{p,k}^{11,k^{'}}$ | 0.75 | Estimated |
| From women | $\beta_{p,k}^{s^{'},1}$ | 1 | Transmission from males to females is assumed to be the same as that from females to males. |
| From uncircumcised men | $\beta_{p,k}^{s^{'},2}$ | 1 |  |
| From circumcised men | $\beta_{p,k}^{s^{'},3}$ | 1 | Assumes no effect of circumcision on  HIV transmission. |
| To circumcised men | $\beta_{p,3}^{s^{'},k^{1}}$ | 0.4 | Risk of HIV acquisition is 60% lower than among uncircumcised men (16-18) |
| Condom efficacy | $\beta_{p,k}^{s^{'},k^{'}}$ | 0.1 | Assumes condoms provide 90% protection from HIV infection |

***Table S3:*** *Factor increments in transmission probability per sex act with respect to baseline transmission probability (β_0_).*

# 1.5 Male circumcision

The level of circumcision changes over time to reflect the increase in male circumcision as has occurred in recent years according to nationally representative surveys and is projected to continue increasing in the future (19-22) (Figure S2) . The movement of uncircumcised men to circumcised classes is represent by the function $g\left( k \right)$, included in the equations below.

$$g\left( 1 \right)=0$$

$$g\left( 2 \right)= \frac{dX_{l,2,a}^{0,p}}{dt}-\eta_{C}$$

$$g\left( 3 \right)= \frac{dX_{l,3,a}^{0,p}}{dt}+\eta_{C}$$

(14)

The parameter $\eta_{C}$ gives the scale-up rate for male circumcision, which is a time-varying parameter based on the extent to which the current level of circumcision in the sexually active adult population matches the data on circumcision prevalence. Movement from uncircumcised to circumcised classes occurs at age 15 to represent circumcision that has occurred after birth but before entering the sexually active population. In the model, the rate of HIV acquisition for circumcised men is reduced by 60% (16-18).

***Figure S2.*** *The proportion of adult men that are circumcised with respect to time.* *The level of circumcision in the model was calibrated to data reported in nationally representative surveys.*

# 1.6 Antiretroviral Treatment

ART can be initiated for the population with four programme types, specified with different initiation rules, as described above. A drop-out rate of 0.005 is assumed, regardless of the CD4 level at which ART is initiated. A rate of progressing to AIDS of 0.105 is assumed for those initiating ART below 200 cells/μL. The survival probability (p) of 0.9 reported by Mahy et. al. (23) was converted to a per capita mortality rate (r), using: p =1 - e^-rt^ . A rate of progressing to AIDS of 0.013 is assumed for those initiating ART above 200 cells/μL. A crude death rate of 1.3 deaths per 100 person years had been reported among individuals receiving early ART in the USA and Canada (24). The number of individuals receiving ART is calibrated to the total number of people on ART in each country modelled (25) (Figure S3).

***Figure S3.*** *The number of adults receiving antiretroviral therapy in Uganda.* *Model data is compared to estimates of the number of adults on ART in (A) South Africa, (B) Malawi, (C) Zimbabwe, and (D) Uganda (25).*

# 1.7 Model calibration

The behavioural parameters (contact rates, proportion of individuals in each risk group, the degree of mixing between risk groups), the baseline transmission probability, and were calibrated as these are difficult to empirically estimate reliably. For each country, the model was calibrated to age- and sex-stratified population size over time (Figures S4-S7), proportion and number on ART (Figure S3), and HIV prevalence (Figure S8).

***Figure S4. Population pyramids for South Africa for 1985, 1990, 1995, 2000, 2005 and 2010.*** *Model population structure is compared to annual age-structured population size model estimates produced by DHS Surveys.*

**

***Figure S5. Population pyramids for Malawi for 1990, 1995, 2000, 2005 and 2010.*** *Model population structure is compared to annual age-structured population size model estimates produced by DHS Surveys.*

**

***Figure S6. Population pyramids for Zimbabwe for 1990, 1995, 2000, 2005 and 2010.*** *Model population structure is compared to annual age-structured population size model estimates produced by DHS Surveys.*

******

***Figure S7. Population pyramids for Zimbabwe for 1985, 1990, 1995, 2000, 2005 and 2010.*** *Model population structure is compared to annual age-structured population size model estimates produced by DHS Surveys.*

***Figure S8. HIV prevalence with respect to time in (A) South Africa, (B) Malawi, (C) Zimbabwe, and (D) Uganda.*** *Prevalence was calibrated to estimates from UNAIDS (25).*

# 1.8 Limitations

The model does not include prevention of mother-to-child transmission (PMTCT), any potential effect of HIV or ART status on the risk of acquiring or dying from COVID-19, or any long-term co-morbidities associated with HIV infection. When individuals re-initiate ART following a disruption due to COVID-19 mitigation or suppression activities, they are assumed to return to the previous HIV stage and ART status as pre-disruption, and the model does not assume any long-term effect on survival due to the disruption, nor any increase in drug resistance.

2. Mortality Risk Sensitivity Analyses

A break in ART for individuals on ART is the primary contributor to the number of excess HIV-related deaths that might occur as a result of COVID-19 mitigation or suppression activities. However, because mortality risk for individuals experiencing a break in ART use is not well known, we modelled three different scenarios of average mortality risk as a sensitivity analysis. In the model, when individuals experience a break in ART, they instantaneously transition to the ‘Treatment dropout’ compartment. In this compartment, individuals survive an average of 10 months prior to death. We used three different values for the monthly risk of mortality for individuals stopping ART during any disruption to ART supply: 0.10%, 0.24%, and 0.44%. The median mortality assumption (0.24% mortality per month) represents a mortality rate similar to that observed in the SMART trial, in which 3% of individuals stopping ART died by 12 months (10), whereas the lower and upper bounds represent hypothetical scenarios in which mortality was substantially lower or higher, respectively.

3 References

1. Cremin I, Alsallaq R, Dybul M, Piot P, Garnett G, Hallett TB. The new role of antiretrovirals in combination HIV prevention: a mathematical modelling analysis. AIDS. 2013;27(3):447-58.

2. Smith JA, Anderson SJ, Harris KL, McGillen JB, Lee E, Garnett GP, et al. Maximising HIV prevention by balancing the opportunities of today with the promises of tomorrow: a modelling study. Lancet HIV. 2016;3(7):e289-96.

3. Beacroft L, Smith JA, Hallett TB. What impact could DMPA use have had in South Africa and how might its continued use affect the future of the HIV epidemic? J Int AIDS Soc. 2019;22(11):e25414.

4. Anderson R, May R. Infectious diseases of humans: dynamics and control. Oxford: Oxford University Press; 1991.

5. Garnett GP, Anderson RM. Factors controlling the spread of HIV in heterosexual communities in developing countries: patterns of mixing between different age and sexual activity classes. Philos Trans R Soc Lond B Biol Sci. 1993;342(1300):137-59.

6. Garnett GP, Anderson RM. Sexually transmitted diseases and sexual behavior: insights from mathematical models. J Infect Dis. 1996;174 Suppl 2:S150-61.

7. Hollingsworth TD, Anderson RM, Fraser C. HIV-1 transmission, by stage of infection. J Infect Dis. 2008;198(5):687-93.

8. Donnell D, Baeten JM, Kiarie J, Thomas KK, Stevens W, Cohen CR, et al. Heterosexual HIV-1 transmission after initiation of antiretroviral therapy: a prospective cohort analysis. Lancet. 2010;375(9731):2092-8.

9. Cohen MS, Chen YQ, McCauley M, Gamble T, Hosseinipour MC, Kumarasamy N, et al. Prevention of HIV-1 infection with early antiretroviral therapy. N Engl J Med. 2011;365(6):493-505.

10. Strategies for Management of Antiretroviral Therapy Study G, El-Sadr WM, Lundgren J, Neaton JD, Gordin F, Abrams D, et al. CD4+ count-guided interruption of antiretroviral treatment. N Engl J Med. 2006;355(22):2283-96.

11. Lodi S, Phillips A, Touloumi G, Geskus R, Meyer L, Thiebaut R, et al. Time from human immunodeficiency virus seroconversion to reaching CD4+ cell count thresholds <200, <350, and <500 Cells/mm(3): assessment of need following changes in treatment guidelines. Clin Infect Dis. 2011;53(8):817-25.

12. United Nations. World Population Prospects 2020 [cited 2020 22 April]. Available from: <http://population.un.org/wpp/>

13. Boily MC, Baggaley RF, Wang L, Masse B, White RG, Hayes RJ, et al. Heterosexual risk of HIV-1 infection per sexual act: systematic review and meta-analysis of observational studies. Lancet Infect Dis. 2009;9(2):118-29.

14. Wawer MJ, Gray RH, Sewankambo NK, Serwadda D, Li X, Laeyendecker O, et al. Rates of HIV-1 transmission per coital act, by stage of HIV-1 infection, in Rakai, Uganda. J Infect Dis. 2005;191(9):1403-9.

15. Ahmed K, Baeten JM, Beksinka M, Bekker L-G, Bukusi EA, Donnell D, et al. HIV incidence among women using intramuscular depot medroxyprogesterone acetate, a copper intrauterine device, or a levonorgestrel implant for contraception: a randomised, multicentre, open-label trial. The Lancet. 2019.

16. Bailey RC, Moses S, Parker CB, Agot K, Maclean I, Krieger JN, et al. Male circumcision for HIV prevention in young men in Kisumu, Kenya: a randomised controlled trial. Lancet. 2007;369(9562):643-56.

17. Auvert B, Taljaard D, Lagarde E, Sobngwi-Tambekou J, Sitta R, Puren A. Randomized, controlled intervention trial of male circumcision for reduction of HIV infection risk: the ANRS 1265 Trial. PLoS Med. 2005;2(11):e298.

18. Gray RH, Kigozi G, Serwadda D, Makumbi F, Watya S, Nalugoda F, et al. Male circumcision for HIV prevention in men in Rakai, Uganda: a randomised trial. Lancet. 2007;369(9562):657-66.

19. National Department of Health (NDoH) S, South Africa (Stats SA) SAMRCS, and ICF,. South Africa Demographic and Health Survey 2016. 2019.

20. National Statistical Office (NSO) [Malawi] and ICF. Malawi Demographic and Health Survey 2015-16. Zomba, Malawi, and Rockville, Maryland, USA: NSO and ICF; 2017.

21. Zimbabwe National Statistics Agency and ICF International. Zimbabwe Demographic and Health Survey 2015: Final Report. Rockville, Maryland, USA: Zimbabwe National Statistics Agency (ZIMSTAT) and ICF International; 2016.

22. Uganda Bureau of Statistics (UBOS) and ICF. Uganda Demographic and Health Survey, 2016. Kampala, Uganda and Rockville, Maryland, USA: UBOS and ICF 2012.

23. Mahy M, Lewden C, Brinkhof MW, Dabis F, Tassie JM, Souteyrand Y, et al. Derivation of parameters used in Spectrum for eligibility for antiretroviral therapy and survival on antiretroviral therapy. Sex Transm Infect. 2010;86 Suppl 2:ii28-34.

24. Kitahata MM, Gange SJ, Abraham AG, Merriman B, Saag MS, Justice AC, et al. Effect of early versus deferred antiretroviral therapy for HIV on survival. N Engl J Med. 2009;360(18):1815-26.

25. UNAIDS. AIDSinfo 2020 [accessed 18 May 2020]. <http://aidsinfo.unaids.org/>
